# Determining the clinical significance of co-colonization of vancomycin-resistant enterococci and methicillin-resistant *Staphylococcus aureus* in the intestinal tracts of patients in intensive care units: a case–control study

**DOI:** 10.1186/s12941-019-0327-8

**Published:** 2019-10-10

**Authors:** Young Kyung Yoon, Min Jung Lee, Yongguk Ju, Sung Eun Lee, Kyung Sook Yang, Jang Wook Sohn, Min Ja Kim

**Affiliations:** 1 Division of Infectious Diseases, Department of Internal Medicine, Korea University Anam Hospital, Korea University College of Medicine, Inchon-ro 73, Seongbuk-gu, Seoul, 02841 Republic of Korea; 2 Infection Control Unit, Korea University Medical Center, Seoul, Republic of Korea; 3 Institute of Emerging Infectious Diseases, Korea University College of Medicine, Seoul, Republic of Korea; 4 Department of Biostatistics, Korea University College of Medicine, Seoul, Republic of Korea

**Keywords:** Methicillin-resistant *Staphylococcus aureus*, Vancomycin-resistant enterococci, Active surveillance, Risk factor, Infection control

## Abstract

**Background:**

The emergence of vancomycin-resistant *Staphylococcus aureus* (VRSA) has become a global concern for public health. The proximity of vancomycin-resistant enterococcus (VRE) and methicillin-resistant *S. aureus* (MRSA) is considered to be one of the foremost risk factors for the development of VRSA. This study aimed to determine the incidence, risk factors, and clinical outcomes of intestinal co-colonization with VRE and MRSA.

**Methods:**

A case–control study was conducted in 52-bed intensive care units (ICUs) of a university-affiliated hospital from September 2012 to October 2017. Active surveillance using rectal cultures for VRE were conducted at ICU admission and on a weekly basis. Weekly surveillance cultures for detection of rectal MRSA were also conducted in patients with VRE carriage. Patients with intestinal co-colonization of VRE and MRSA were compared with randomly selected control patients with VRE colonization alone (1:1). Vancomycin minimum inhibitory concentrations (MICs) for MRSA isolates were determined by the Etest.

**Results:**

Of the 4679 consecutive patients, 195 cases and 924 controls were detected. The median monthly incidence and duration of intestinal co-colonization with VRE and MRSA were 2.3/1000 patient-days and 7 days, respectively. The frequency of both MRSA infections and mortality attributable to MRSA were higher in the case group than in the control group: 56.9% vs. 44.1% (*P *= 0.011) and 8.2% vs. 1.0% (*P* = 0.002), respectively. Independent risk factors for intestinal co-colonization were enteral tube feeding (odds ratio [OR], 2.09; 95% confidence interval [CI] 1.32–3.32), metabolic diseases (OR, 1.75; 95% CI 1.05–2.93), male gender (OR, 1.62; 95% CI 1.06–2.50), and Charlson comorbidity index < 3 (OR, 3.61; 95% CI 1.88–6.94). All MRSA isolates from case patients were susceptible to vancomycin (MIC ≤ 2 mg/L).

**Conclusions:**

Our study indicates that intestinal co-colonization of VRE and MRSA occurs commonly among patients in the ICU with MRSA endemicity, which might be associated with poor clinical outcomes.

## Background

Over the past decade, methicillin-resistant *Staphylococcus aureus* (MRSA) and vancomycin-resistant enterococcus (VRE) have been endemic in hospital settings throughout the world. According to a recent nationwide surveillance study, the prevalence rates of MRSA and vancomycin-resistant *Enterococcus faecium* (VREF) in the Republic of Korea (ROK) were 66–72% and 29–31% from 2013 to 2015, respectively [1].

Since 1961 when British scientists discovered MRSA infection, vancomycin has been regarded as the standard option for initial treatment of MRSA infections for more than half a century. However, the accelerated use of vancomycin since the mid-1980s resulted in the emergence of MRSA with reduced susceptibility to vancomycin [2]. Particularly, vancomycin minimum inhibitory concentration (MIC) creep for MRSA isolates, probably associated with poorer clinical outcomes, has become a major worldwide concern [3, 4]. Although inconsistent information about the MIC creep phenomenon and conflicting results have been noted [5, 6], the emergence of vancomycin-intermediate *S. aureus* (VISA) since 1997 and vancomycin-resistant *S. aureus* (VRSA) since 2002 has become a global challenge [7, 8].

The mechanisms by which *S. aureus* isolates become more resistant to vancomycin are not established. To date, there is no evidence that the VISA isolates have the *van* genes found in VRE. However, recently there has been in vitro transfer of the *vanA* gene from *Enterococcus faecium* to *S. aureus* [9], and VRSA isolates containing the *vanA* gene were isolated from the patients, probably acquired by *S. aureus* from VRE [10]. Of note, the clinical specimens of those patients were co-colonized with MRSA and VRE [10, 11]. Furthermore, the *vanA* gene isolated from the VRSA strain was identical to the *vanA* gene present in *Enterococcus faecalis* cultured from the same patient [10].

The spread of vancomycin resistance occurs through not only clonal transmission of enterococcus strains between patients but also plasmid and transposon dissemination of resistance determinants between Gram-positive bacteria of different genera [12]. Indeed, previous studies have suggested that the proximity of VRE and MRSA may be one of the foremost risk factors for the development of VRSA [13, 14]. In that context, several reports investigated the epidemiology and clinical features of patients co-colonized or coinfected with VRE and MRSA concurrently, isolated from the different specimens acquired by active surveillance or clinical cultures [15–21].

This study aimed to examine the incidence, risk factors, and clinical outcomes of concurrent co-colonization with VRE and MRSA in the intestinal tracts of patients in the intensive care units (ICUs) where MRSA is highly endemic.

## Methods

### Study design and patients

This case–control study was performed in 52-bed ICUs in a 1051-bed university-affiliated hospital in the ROK between September 2012 and October 2017. Active surveillance using rectal cultures for VRE were conducted on a weekly basis and at ICU admission. Weekly surveillance cultures for detection of rectal MRSA were also conducted in patients with VRE carriages. Cases with intestinal co-colonization of VRE and MRSA were compared in a 1:1 ratio with randomly selected control patients with a positive active surveillance culture for VRE and a negative one for MRSA.

To monitor the prevalence of MRSA carriage, the hospital ran a program of active surveillance of nasal cultures at ICU admission for MRSA acquisition in all patients who stayed in the ICUs for more than 24 h during the study period.

The Institutional Review Board (IRB) of the Korea University Anam Hospital approved the protocol and waived the need for informed consent (IRB registration no. 2017AN0823).

### Data collection and definitions

The patients with both VRE and MRSA, determined from active surveillance using their rectal specimens, were assigned to the case group, and those with only VRE without MRSA were assigned to the control group. In the case group, the interval between identification of specimens positive for VRE and MRSA was within 1 week.

Clinical data were collected from patients in the case and control groups from September 2012 to October 2017. Electronic medical records were reviewed to collect relevant demographic and clinical information: age, gender, date of hospital admission, admission route (emergency room or outpatient setting), comorbidities, Charlson comorbidity index [22], microbiological data, in-hospital mortality and recent exposure to medical procedures, recent surgery, or antimicrobial drugs taken within the last 90 days.

### Microbiological evaluation

Isolation and detection of VRE from rectal swab samples were carried out as described previously [23]. Since 2017, rectal swabs for screening VRE were plated directly onto chromID VRE-Select agar plates (bioMérieux, Marcy l’Etoile, France). To screen for the presence of MRSA, perirectal swabs or nasal swabs were inoculated directly onto chromID MRSA-Select agar plates (bioMérieux, Marcy l’Étoile, France) and incubated overnight at 35 °C. We also analyzed microbiological data from clinical cultures of all the case and control patients that were selected for clinical diagnosis of infectious diseases at the discretion of a physician in routine clinical practice.

Species identification and drug susceptibility testing of isolates were performed using the VITEK 2 (bioMèrieux, Hazelwood, MO) system or MicroScan WalkAway 96 plus (Siemens Healthcare Diagnostics Inc., CA, USA) system based on the standard criteria defined by the Clinical and Laboratory Standards Institute [24].

MRSA isolates collected from patients with intestinal co-colonization of VRE and MRSA since October 2015 were available for evaluation of vancomycin minimum inhibitory concentrations. For MRSA isolates collected from patients in the case group, Etest analysis of vancomycin and teicoplanin were performed using Etest strips (bioMérieux, Marcy l’Etoile, France).

### Statistical analysis

For descriptive purposes, categorical and continuous variables were calculated as frequencies (proportion) and median (interquartile ranges [IQR]), respectively. In the univariate analyses, the Pearson’s Chi square test or Fisher’s exact test was used to compare groups of categorical variables. A two-sample Student’s *t* test or the Mann–Whitney *U* test was used to compare groups of normally or non-normally distributed continuous variables, respectively.

Multivariate logistic regression analysis using backward stepwise variable selection based on the Wald statistic was used to identify risk factors associated with intestinal co-colonization with VRE and MRSA. The models were evaluated using the Hosmer–Lemeshow goodness-of-fit test. The predictive accuracy of the final logistic regression model was calculated using leave-one-out cross-validation (LOOCV). Receiver-operating curves were also constructed for the multivariate logistic regression model to evaluate discriminable predictability between case and control groups.

A linear regression model using measures on a per-month basis was used to determine the trends in the square-root transformed median monthly incidence of intestinal co-colonization with VRE and MRSA. The Durbin-Watson’s statistic d value for the transformed dependent variable was 1.92 for study data. The Durbin-Watson’s statistic d value of nearly 2.0 indicates that there is no autocorrelation.

IBM SPSS Statistics version 20.0 (IBM Corporation, Armonk, NY, USA), SAS 9.4 (SAS Institute Inc., Cary, NC, USA), and R 3.5.2 (The R Foundation for Statistical Computing, Vienna, Austria) were used for all statistical analyses. Two-sided *P* values < 0.05 were considered significant.

## Results

### Epidemiology of intestinal co-colonization with VRE and MRSA

From September 2012 to October 2017, surveillance cultures for rectal VRE were obtained from 4679 patients and disclosed 5410 VRE-positive samples (37.2%) out of 14,548 rectal swabs from ICU patients; this accounted for 1273 patients. Of VRE isolates, 98.7% (n = 1257) were *E. faecium*, 1.0% (n = 13) were *E. faecalis*, and 0.2% (n = 3) were both. The proportion of VRE was 50.6% (n = 7885) of all enterococcal isolates (n = 15,595), and the median monthly incidence of VRE acquisition was 7.3 (IQR, 4.0–10.1; range, 0–18.1)/1000 patient-days. Of all staphylococcal isolates (n = 15,276), 71.0% (n = 10,846) were MRSA, and the median monthly incidence of MRSA was 9.5 (IQR, 7.4–14.4; range, 3.3–21.4)/1000 patient-days. In active surveillance using a nasal swab for MRSA, there was a significant difference in the frequency of MRSA nasal carriage between the case group and control group (16.4% vs. 0, *P *< 0.001).

**Fig. 1:**
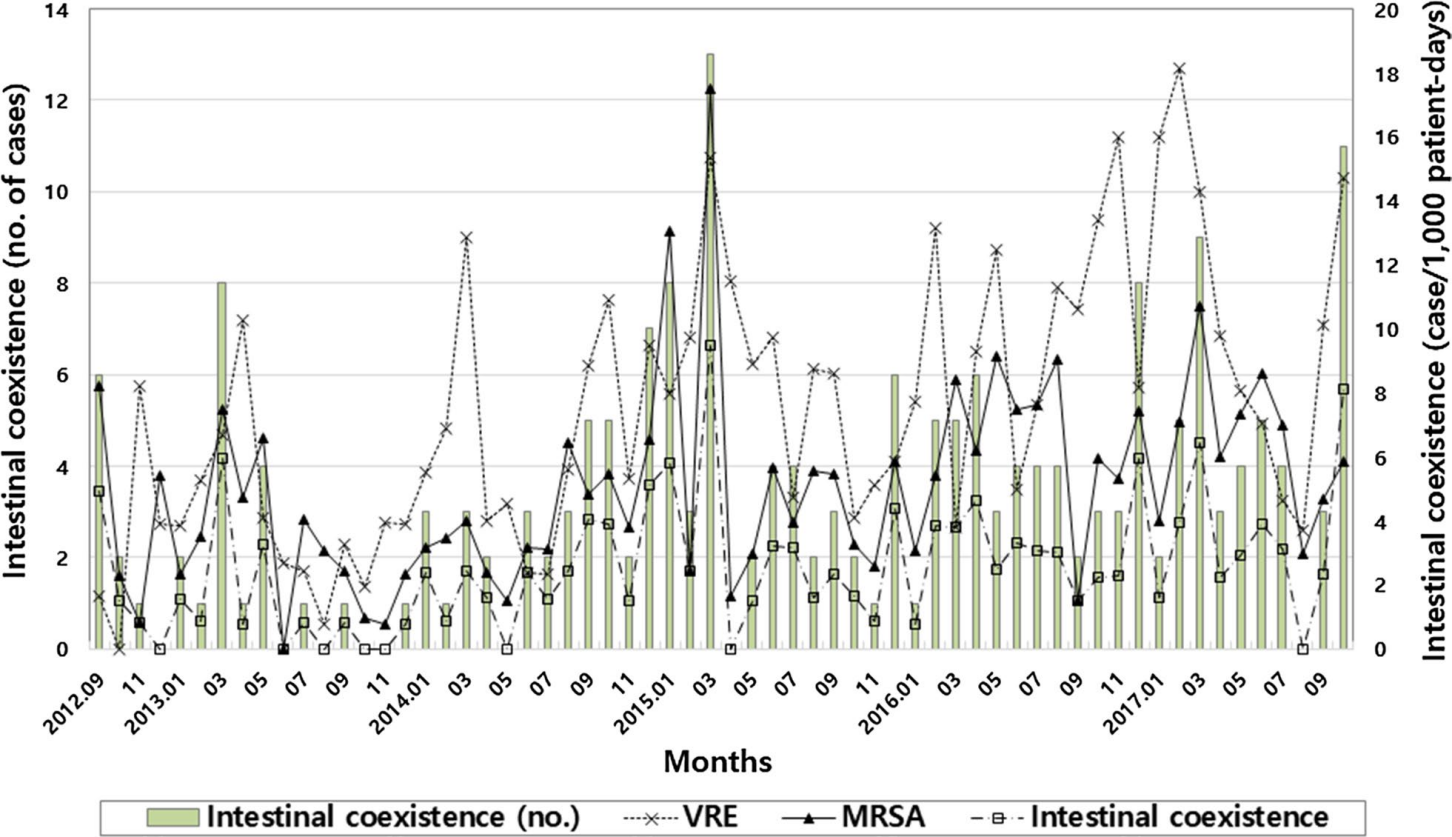
Monthly incidence of vancomycin-resistant enterococci (VRE) or intestinal co-colonization of VRE and methicillin-resistant *Staphylococcus aureus* (MRSA) (cases per 1000 patient-days) and number of intestinal co-colonization of VRE and MRSA cases in the intensive care units from September 2012 to October 2017

Of 1273 patients colonized with VRE, 195 cases (15.3%) with intestinal co-colonization of VRE and MRSA and 924 controls who were positive for VRE and negative for MRSA were identified. In the patients colonized with VRE, the median monthly incidence of intestinal co-colonization was 2.3/1000 patient-days (IQR, 0.9–3.8; range, 0.0–9.5) (Fig. 1). In the linear regression model, the square-root transformed median monthly incidence of intestinal co-colonization with VRE and MRSA showed a significantly increased trend (coefficient for time = 0.015, *P* = 0.003; Fig. 1). The median duration of an ICU stay before intestinal co-colonization with VRE and MRSA was 21 days (IQR, 14–32; range, 1–220). The baseline demographic and clinical characteristics of the patients with intestinal co-colonization with VRE and MRSA are shown in Table 1.

**Table 1:** Comparison of demographic and clinical characteristics between the case group and the control group

Characteristics	Total (n = 390)	Cases (n = 195)	Controls (n = 195)	*P* value
Demographic variable				
Median age, years (IQR)	72 (59–79)	72 (63–79)	71 (58–78)	0.183
Male sex, n (%)	176 (45.1)	98 (50.3)	78 (40.0)	0.042
Admission route				0.046
Emergency room	97 (24.9)	40 (20.5)	57 (29.2)	
Outpatient setting	293 (75.1)	155 (79.5)	138 (70.8)	
Variables from current admission				
Median length of hospital stay before VRE acquisition, days (IQR)	21 (14–32)	21 (14–32)	46 (27–77)	0.522
MRSA nasal carriage, n (%)	32 (8.2%)	32 (16.4%)	0	< 0.001
Comorbidities, n (%)				
Cardiovascular diseases	233 (59.7)	119 (61.0)	114 (58.5)	0.606
Neurologic diseases	84 (21.5)	39 (20.0)	45 (23.1)	0.460
Malignancy diseases	64 (16.4)	29 (14.9)	35 (17.9)	0.412
Renal diseases	47 (12.1)	26 (13.3)	21 (10.8)	0.427
Hepatic diseases	36 (9.2)	15 (7.7)	21 (10.8)	0.294
Pulmonary diseases	43 (11.0)	15 (7.7)	28 (14.4)	0.036
Metabolic diseases	132 (33.8)	70 (35.9)	62 (31.8)	0.392
Hematologic diseases	4 (1.0)	0	4 (2.1)	0.123
Median Charlson comorbidity score (IQR)	2 (1–2)	1 (1–2)	2 (1–3)	0.020
Charlson comorbidity score ≥ 3, n (%)	82 (21.0)	28 (14.4)	54 (27.7)	0.001
Exposure to antimicrobial in the previous 90 days, n (%)				
Vancomycin	161 (41.3)	81 (41.5)	80 (41.0)	0.918
Imipenem	170 (43.6)	92 (47.2)	78 (40.0)	0.153
Cephalosporins	225 (57.7)	122 (62.6)	103 (52.8)	0.051
Fluoroquinolones	196 (50.3)	106 (54.4)	90 (46.2)	0.105
Procedures in the previous 90 days, n (%)				
Urinary catheter	326 (83.6)	171 (87.7)	155 (79.5)	0.029
Enteral feeding tube	254 (65.1)	144 (73.8)	110 (56.4)	< 0.001
Mechanical ventilator	201 (51.5)	116 (59.5)	85 (43.6)	0.002
Prior ICU admission	356 (91.3)	184 (94.4)	172 (88.2)	0.031
Prior operative procedure	221 (56.1)	105 (53.8)	116 (59.5)	0.261
Positive clinical culture during current admission, n (%)				
MRSA positive	162 (41.5)	114 (58.5)	48 (24.6)	< 0.001
VRE positive	55 (14.1)	23 (11.8)	32 (16.4)	0.190
Co-isolation of VRE and MRSA	23 (5.9)	17 (8.7)	6 (3.1)	0.032
Episodes of infections during current admission, n (%)				
VRE infection	14 (3.6)	5 (2.6)	9 (4.6)	0.276
MRSA infection	197 (50.5)	111 (56.9)	86 (44.1)	0.011
Clinical outcomes				
In-hospital mortality, n (%)	93 (23.8)	48 (24.6)	45 (23.1)	0.721
Attributable mortality, n (%)				
MRSA	18 (4.6)	16 (8.2)	2 (1.0)	0.002
VRE	0	0	0	–
Length of hospital stay after VRE acquisition, median (IQR), days	18 (6–46)	21 (7–47)	14 (5–46)	0.089

*ICU* intensive care unit, *IQR* interquartile range, *MRSA* methicillin-resistant *Staphylococcus aureus*, *VRE* vancomycin-resistant enterococci

The median duration of intestinal co-colonization with VRE and MRSA was 7 days (IQR, 7–14; range, 7–70). The duration by the end-of-surveillance culture was distributed as follows: 1 week (number of total cases = 136; number with persistent co-colonization on discharge = 27), 2 weeks (27; 4), 3 weeks (20; 4), 4 weeks (7; 1), 5 weeks (2; 0), 7 weeks (2; 1), and 10 weeks (1; 1).

In patients included in the analysis, the distribution of VRE and MRSA isolated from clinical specimens is demonstrated in Table 2. There was a significant difference in the frequency of co-colonization of VRE and MRSA on subsequent clinical specimens during current admissions between the case group and control group (8.7% vs. 3.1%, *P *= 0.032). Of 23 patients whose clinical specimen showed co-colonization of VRE and MRSA, only 1 (4.3%) had simultaneous isolation of VRE and MRSA in the same specimen submitted for sputum culture.

**Table 2:** Distribution of vancomycin-resistant enterococci (VRE) and methicillin-resistant *Staphylococcus aureus* (MRSA) isolates by clinical specimen

	VRE isolates			MRSA isolates		
Specimen, n (%)	Total	Cases	Controls	Total	Cases	Controls
Negative	335 (85.9)	172 (88.2)	163 (83.6)	228 (58.5)	81 (41.5)	147 (75.4)
Positive	55 (14.1)	23 (11.8)	32 (16.4)	162 (41.5)	114 (58.5)	50 (25.6)
Urine	41 (10.5)	17 (8.7)	24 (12.3)	2 (0.5)	2 (1.0)	0
Wound	1 (0.3)	1 (0.5)	0	7 (1.8)	3 (1.5)	4 (2.1)
Blood	7 (1.8)	3 (1.5)	4 (2.1)	13 (3.3)	5 (2.6)	8 (4.1)
Bile	1 (0.3)	0	1 (0.5)	0	0	0
Sputum	1 (0.3)	0	1 (0.5)	128 (32.8)	95 (48.7)	33 (16.9)
Tip of drainage catheter	2 (0.5)	1 (0.5)	1 (0.5)	3 (0.8)	1 (0.5)	2 (1.0)
Others*	2 (0.5)	1 (0.5)	1 (0.5)	9 (2.3)	8 (4.1)	1 (0.5)
***Total***	***390 (100)***	***195 (100)***	***195 (100)***	***390 (100)***	***195 (100)***	***195 (100)***

*MRSA* methicillin-resistant *Staphylococcus aureus*, *VRE* vancomycin-resistant enterococci

*Eye discharge, ascites, vaginal swab, pericardial fluid, and pleural fluid

### Risk factors for intestinal co-colonization with VRE and MRSA

Results of bivariate analysis of risk factors for intestinal co-colonization with VRE and MRSA among patients with intestinal colonization with VRE are shown in Table 1. The median age was similar between the two groups (*P *= 0.183); however, the case group had more male patients than the control group (*P *= 0.042). There was no difference in the length of a hospital stay before VRE acquisition between the two groups (*P *= 0.522). The control patients were more likely to have underlying pulmonary diseases than the case patients (*P *= 0.036). Case patients had lower Charlson comorbidity indexes than control patients (*P *= 0.020). There was no difference in the exposure to antimicrobial drugs in the previous 90 days between the two groups, but case patients had been exposed to invasive devices (indwelling urinary catheters, enteral feeding tubes, and mechanical ventilator care) more frequently and were more likely to have a prior ICU admission in the previous 90 days than controls (Table 1).

Multivariate logistic regression analyses demonstrated that exposure to enteral feeding tubes, metabolic diseases, male gender, and a Charlson comorbidity index < 3 were independent risk factors for intestinal co-colonization with VRE and MRSA (Table 3). The *P*-value for the Hosmer–Lemeshow goodness-of-fit test was 0.908, which was greater than the 0.05 significance threshold; therefore, there was no significant evidence for lack of fit in any of the final models.

**Table 3:** Multivariate analysis of risk factors for intestinal co-colonization of methicillin-resistant *Staphylococcus aureus* (MRSA) among patients with intestinal colonization with VRE

Variables	Odds ratio	95% confidence interval	*P*-value
Sex (male)	1.62	1.06–2.50	0.027
Enteral tube feeding (yes)	2.09	1.32–3.32	0.002
Metabolic diseases (yes)	1.75	1.05–2.93	0.032
Charlson comorbidity index < 3	3.61	1.88–6.94	< 0.0001
Prior operative procedure (no)	1.47	0.95–2.27	0.083
Prior ICU admission (yes)	2.03	0.90–4.60	0.089
Chronic renal diseases (yes)	2.00	0.96–4.18	0.064

LOOCV was performed to assess the predictive accuracy of the final model. The areas under the receiver operating characteristic curve for the model were 0.679 (95% CI 0.631–0.725) and 0.638 (95% CI 0.588–0.686) for both raw data and leave-one-out cross-validation. The sensitivity, specificity, positive predictive value, and negative predictive value obtained with an optimal cut-off point are described in Additional file 1: Fig. S1.

### Clinical outcomes

There was no difference in the frequencies of MRSA or VRE bacteremia and VRE infections requiring antibiotic treatment between the two groups (Table 1). However, MRSA infections requiring antibiotic treatment were more common in the case group than in the control group (56.9% vs. 44.1%, *P *= 0.011). Of the case patients, 24.6% died during the current admission. However, no significant difference was observed in in-hospital mortality (*P *= 0.721) and the median length of a hospital stay after VRE acquisition (*P *= 0.089) between the case group and control group (Table 1). Eighteen patients died of MRSA sepsis, while none died of VRE. Moreover, significant differences were noted between cases and controls in terms of the rate of mortality attributable to MRSA (8.2% vs. 1.0%, *P *= 0.002).

### Glycopeptide susceptibility of MRSA isolates

An Etest was performed on the 70 MRSA isolates cultured from the case patients. The vancomycin MIC_50_, MIC_90_, and range by the standard Etest were 1.0, 1.5, and 0.38–2.0 mg/L, and the teicoplanin MIC_50_, MIC_90_, and range by the macro Etest were 3.0, 4.0, and 0.75–6.0 mg/L, respectively. According to the Etest results, no VISA or VRSA was detected among these isolates. There was no significant difference in the frequency of MRSA isolates with vancomycin MIC ≥ 1.0 mg/L [22/24 (91.7%) vs. 36/46 (78.3%), *P *= 0.197) or ≥ 1.5 mg/L [9/24 (37.5%) vs. 16/46 (34.8%), *P *= 0.822) between the patients with intestinal co-colonization of VRE and MRSA maintained for more than 2 weeks and those who maintained for only 1 week.

## Discussion

This study suggested that intestinal co-colonization with VRE and MRSA was not uncommon in 15.3% (195/1273) of ICU patients with rectal colonization of VRE in the hospital setting where MRSA is highly endemic. However, there was no evidence of a correlation between the proximity of VRE and MRSA in the same site of the intestine and the decreased susceptibility to vancomycin of MRSA isolates. Intestinal co-colonization with VRE and MRSA appears to have a negative effect on mortality attributable to MRSA compared with rectal colonization of VRE alone.

In our study, MRSA acquisition adversely affects the frequency of MRSA infections among patients with intestinal co-colonization with VRE and MRSA, which is consistent with findings of previous studies [21]. It is well known that 19–30% of patients colonized with MRSA develop subsequent MRSA infections, and MRSA carriers have an increased risk of up to 9.5-fold for subsequent MRSA infection compared with those not colonized with MRSA [25, 26]. Of note, mortality attributable to MRSA among patients with VRE and MRSA co-colonization was also significantly higher than that due to VRE among the MRSA-negative patients.

Compared with the results of clinical cultures, our data showed that active surveillance resulted in the detection of more unrecognized carriers of both MRSA and VRE and made a substantially larger impact on the assessment of VRE colonization than on MRSA carriage. A few studies have suggested the duration of spontaneous colonization appears to be 45–306 days for VRE and 12–616 for MRSA, although the results varied widely [27–30]. In our analysis, intestinal carriage of both VRE and MRSA could be persistent up to 70 days, which would increase the potential to transmit either or both of these microorganisms to other patients. Therefore, actively identifying patients colonized with MRSA in the intestine is of value among patients colonized with VRE in order to improve the effects of infection prevention and control and clinical outcomes, particularly in high-prevalence clinical settings [31]. However, the results of these surveillance cultures will be more meaningful when effective interventions are developed.

Our study showed that exposure to enteral feeding tubes, metabolic diseases, male gender, and Charlson comorbidity index < 3 were independent risk factors for intestinal co-colonization with VRE and MRSA. In previous studies, independent risk factors for co-colonization or coinfection with VRE and MRSA were age, male gender, prior hospitalization, residing in a long-term care facility, prior ICU admission, exposure to invasive medical devices, renal insufficiency, use of antimicrobial agents, presence of vancomycin-resistant *E. faecalis*, and impaired consciousness [16, 18–20, 32, 33]. In our analysis, use of enteral feeding tube has been significantly associated with intestinal co-colonization of VRE and MRSA. Previous reports found that use of the nasogastric tube has been associated with intestinal colonization with VRE and *Clostridium difficile*, and its use may be the mediator of nasal MRSA transfer to the intestine [34, 35]. In our study, 16.4% of patients who were rectal MRSA carriers also had nasal colonization, similar to the findings of previous studies [34, 36]. Although the pathogenesis is not well defined, decreased gastric acidity and antibiotic effects that eliminate competing microflora may explain the mechanism underlying the occurrence of intestinal colonization in patients who are nasal MRSA carriers. Considering VRSA isolates found on a biofilm within an indwelling catheter where VRE and MRSA strains coexisted [37], it is necessary to minimize the prolonged use of medical devices and monitor the emergence of decreased susceptibility to glycopeptides for MRSA. Although the reason for the Charlson comorbidity index < 3 being the major risk factor is unclear and was not determined by this study, a coincidence was reported in previous studies [16].

Our analysis presented the prevalence of intestinal co-colonization of VRE and MRSA in 1.3% (195/14,489) of the 14,489 ICU patients who underwent rectal VRE culture and 15.3% (195/1273) among 1273 ICU patients with rectal colonization of VRE, respectively. Other studies have also suggested its prevalence ranges from 2.7 to 34.8%; however, these studies were based on surveillance cultures obtained from different sites or clinical cultures [18–20, 32, 33]. The design of these studies was different from that of our study; that is, samples for active surveillance cultures were obtained from the same site in the intestine, in order to identify as many carriers as possible while maintaining close proximity of VRE and MRSA. Although our results showed a significant difference (prevalence: 15.3%), a previous study conducted in a similar setting showed that 54.1% (20/37) of patients whose intestinal tracts were colonized with VRE also had coexisting gastrointestinal colonization with MRSA [38].

This study showed that all MRSA isolates recovered from the case patients who were available for Etests were susceptible to vancomycin and teicoplanin. Although vancomycin-resistant genes are common in VRE isolates and the prevalence of co-colonization with VRE and MRSA is high, the transfer of the mobile genetic elements from VRE to MRSA seemed to be a rare event. Interestingly, *E. faecalis*, containing Inc18 plasmids and Tn1549 transposons, has been more often associated with conjugation events and subsequent VRSA colonization or infection compared with other *Enterococcus* species [9, 10, 14]. Furthermore, *vanA* genes that are resistant to high levels of glycopeptides are more likely to be involved in the transmission of vancomycin resistance to MRSA than *vanB* genes [14, 39, 40]. However, in our study, most VRE isolates were *E. faecium*. Therefore, these findings would have reduced the likelihood of VRSA emergence.

Our study had several limitations. First, this study was performed on a small scale in the ICUs of a single medical center. Consequently, the area under the receiver operating characteristic curve for our model was 0.679, which was lower than expected. There are several possibilities that may account for this finding, including the nonlinear relationships, higher order interactions between variables, or risk factors not evaluated. Therefore, our results may not be applicable to other settings, and larger-scale multicenter studies are required in the future. Second, the study was limited to the intestinal co-colonization of VRE and MRSA. Our study findings should be interpreted with caution, taking into account the possibility that simultaneous colonization of VRE and MRSA isolated from each of the different body parts, in the same patient, would affect the emergence of VRSA. Third, both cases and controls included patients whose intestinal co-colonization or VRE colonization was confirmed only once. Therefore, it is difficult to completely eliminate the possibility of contamination. The median duration of intestinal co-colonization with VRE and MRSA was 7 days; in the majority of case patients (n = 136, 69.7%), VRE and MRSA co-colonization were maintained for only 1 week. A short period of close proximity of VRE and MRSA may have affected the low frequency of VRSA emergence.

## Conclusions

In conclusion, 195 (15.3%) of 1273 patients with rectal colonization of VRE had intestinal co-colonization with VRE and MRSA on active surveillance cultures. Considering its risk factors, minimizing the use of enteral tube feeding would contain the spread of multidrug-resistant bacteria and improve clinical outcomes. In our study, intestinal co-colonization with VRE and MRSA does not result in the emergence of VRSA. However, active surveillance for occurrences of intestinal co-colonization with VRE and MRSA and the emergence of VISA or VRSA is warranted due to its clinical significance and undefined mechanism of vancomycin-resistance transfer.

## Supplementary information

**Additional file 1: Fig. S1.** Receiver operating characteristic curve of the diagnosis of the final penalized logistic regression model for the risk factors for intestinal co-colonization with vancomycin-resistant enterococci and methicillin-resistant *Staphylococcus aureus* in ICU patients.

## Data Availability

Data generated or analyzed during this study are available. Further information and requests for data sharing will be fulfilled by contacting the first author, Young Kyung Yoon (young7912@korea.ac.kr).

## Abbreviations

AUC: area under the receiver operating characteristic curve
CI: confidence interval
VISA: vancomycin-intermediate *Staphylococcus aureus*
ICU: intensive care unit
IQR: interquartile ranges
IRB: institutional review board
LOOCV: leave-one-out cross validation
MIC: minimum inhibitory concentration
MRSA: methicillin-resistant *Staphylococcus aureus*
NPV: negative predictive value
PPV: positive predictive value
ROK: Republic of Korea
VRE: vancomycin-resistant enterococcusVREF, vancomycin-resistant *Enterococcus faecium*
VRSA: vancomycin-resistant *Staphylococcus aureus*
