# Supplementary figures and images for: Determining the clinical significance of co-colonization of vancomycin-resistant enterococci and methicillin-resistant Staphylococcus aureus in the intestinal tracts of patients in intensive care units: a case–control study
Source: Ann Clin Microbiol Antimicrob. 2019 Oct 10;18:28. doi: 10.1186/s12941-019-0327-8 (PMC6785887; doi:10.1186/s12941-019-0327-8)

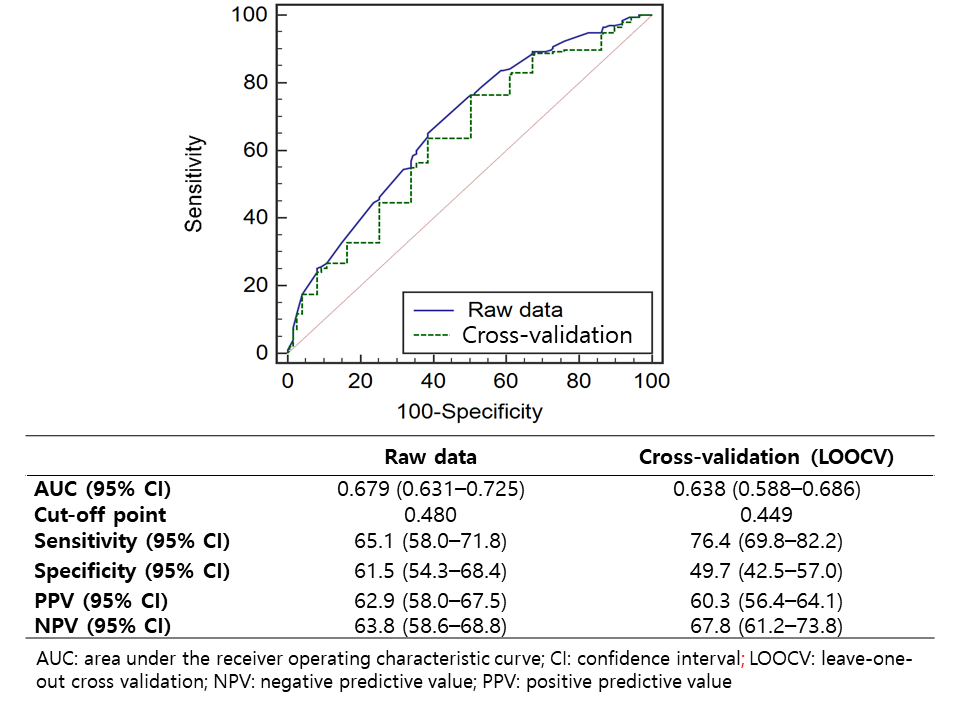

Supplement: Supplementary file 1 — Additional file 1: Fig. S1. Receiver operating characteristic curve of the diagnosis of the final penalized logistic regression model for the risk factors for intestinal co-colonization with vancomycin-resistant enterococci and methicillin-resistant Staphylococcus aureus in ICU patients. [file 12941_2019_327_MOESM1_ESM.tiff]
